# Biaxial flexural strength of 3D-printed 3Y-TZP zirconia using a novel ceramic printer

**DOI:** 10.1007/s00784-024-05533-5

**Published:** 2024-02-13

**Authors:** Andreas Zenthöfer, Ali Ilani, Clemens Schmitt, Peter Rammelsberg, Sebastian Hetzler, Stefan Rues

**Affiliations:** Department of Prosthodontics, University Clinic Heidelberg, University of Heidelberg, 69120 Heidelberg, Germany

**Keywords:** 3D printing, Zirconia, Flexural strength, Additive manufacturing, Ceramics

## Abstract

**Objectives:**

To compare the strength and reliability of 3D-printed 3Y-TZP zirconia manufactured with various printing orientations and staining.

**Materials and methods:**

A total of one-hundred cylindrical zirconia specimens were designed and fabricated using 3D printing and processed according to ISO 6872 standards. Of these specimens, 80 were 3D printed using the new ZIPRO-D (ZD) 3D ceramic printer. In this ZD group, 60 specimens were printed in a vertical orientation and were either stained after debinding (ZD1, x-orientation, *n* = 20) or not stained (ZD2, x-orientation, *n* = 20; ZD3, y-orientation, *n* = 20) and the remaining 20 specimens out of *n* = 80 were printed in a horizontal orientation (ZD4). Further 20 specimens out of the entire sample *N* = 100 were printed vertically with the CeraFab7500 3D ceramic printer (LC). All completed specimens were loaded until fracture using a universal testing machine. Biaxial flexural strengths and Weibull parameters were computed for the ZD groups and for the LC group. Group and sub-group effects were evaluated using Welch ANOVA (alpha = 0.05).

**Results:**

The mean (standard deviation, SD) biaxial flexural strengths of vertically oriented ZD samples with (ZD1) and without (ZD2/ZD3) staining were 811 (197) and 850 (152) MPa, respectively (*p* > 0.05). The ZD4 (horizontally printed), 1107 (144) MPa, and LC (1238 (327)) MPa samples had higher mean (SD) flexural strengths than the ZD1–3 specimens. No difference was observed between the ZD4 and LC group (*p* > 0.05). Weibull moduli were between *m* = 4.6 (ZD1) and 9.1 (ZD4) in the ZD group and *m* = 3.5 in the LC group.

**Conclusions:**

All tested 3D-printed zirconia specimens exceeded the flexural strengths required for class 5 restorations according to ISO 6872 standards. While the flexural strengths of zirconia printed using the novel ZD device in the vertical orientation are lower than those of zirconia printed using the LC printer, the ZD printer shows at least comparable reliability.

**Clinical relevance:**

3D-printing of zirconia is a new technology in dental application. Based on the presented strengths values, clinical application of 3D-printed zirconia for fixed dental protheses can be recommended.

## Introduction

Zirconia materials have become more and more popular for various dental indications. Zirconia offers esthetical advantages and better biocompatibility compared with metal-based restorations while material properties are favorable [1, 2]. In addition, translucent zirconia modifications have been developed for minimally invasive monolithic or partially veneered restorations that can reduce the loss of tooth substance [3]. Zirconia restorations are almost exclusively fabricated by milling according to a CAD-CAM workflow. Some years ago, additive lithography-based ceramic manufacturing (LCM) was introduced.

Additive manufacturing of dental restorations using printing technology rather than milling has gained interest because it can create thinner and finer structures by avoiding tooling stress and milling radius correction [4–8]. The Organization for Economic Cooperation and Development (OECD) speculates that additive manufacturing will replace subtractive approaches in the next few years [9]. Schweiger and co-workers take a similar view; in their review, a great potential is attributed to DLP printing in dental applications. In particular, the manufacturing approach of a 3D-printer by the Austrian company Lithoz is described as forward-looking [8]. However, the fit, strength, and reliability of restorations made with these new technologies must be measured with those of restorations made by milling.

In LCM, dental restorations are built up from a resin-zirconia dispersion layer by layer, each following photopolymerization. Completed objects are cleaned with isopropanol alcohol, debinded from resin parts, and sintered. This, and the printing process, can create flaws or voids that do not occur with milling. Delaminations between the printed layers may also occur [4, 5, 10–12]. Previous reports investigating the biaxial flexural strength of 3D-printed zirconia specimens have found smaller Weibull parameters, indicating lower reliability, compared with milled variants, which is not surprising considering the potential material flaws [13–16]. Out of these studies Zenthöfer and co-workers (2022) as well as Bergler and co-workers (2021) used the Lithoz CeraFab 7500 3D-printer, LC (14, 16). A further study investigating flexural strength of 3D-printed restorations made by LC found that prolonged cleaning damaged the specimens, dramatically reducing their reliability [17].

Since LCM technology was introduced, various optimizations and adjustments have been made to the materials and workflow. LC-made restorations offer clinically acceptable marginal and internal fit [18]; the performance seems to be still inferior in some aspects to that of milled restorations while biocompatibility [19] is comparable. Recently, a new 3D printer using digital light processing technology was developed—the ZIPRO-D (ZD) printer. The ceramic slurry and workflow of the ZD printer have been certified by the FDA, CE and ISO, so it is now suitable for clinical use. Objects are printed on the platform from the bottom to the top, which should guarantee torsion freeness. However, the biaxial flexural strength and Weibull parameters of these 3D-printed zirconia restorations have not been systematically evaluated. The layered printing process may still cause the ceramics to behave anisotropically after debinding and sintering.

The aim of this laboratory study was to evaluate the biaxial flexural strength and reliability of zirconia printed on the ZD printer using different nesting orientations (vertical and horizontal) and staining or no staining after debinding. We also compared the material properties of zirconia fabricated by the ZD printer with those of zirconia made by the LC printer, which has been used in previous studies. The microstructure of the 3D-printed specimens was also evaluated by light and scanning electron microscopy (SEM).

The study hypotheses were (1) staining of specimens will significantly influence the flexural strength of printed zirconia, (2) printing orientation will significantly affect flexural strength, and (3) there will be significant differences between specimens printed using the ZD and LC printer.

## Materials and methods

### Setting and sampling

**Fig. 1:**
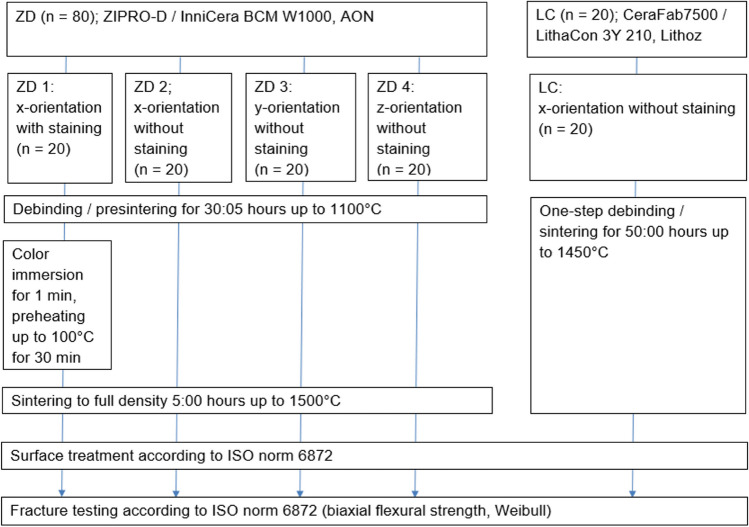
Sampling and study workflow

**Fig. 2:**
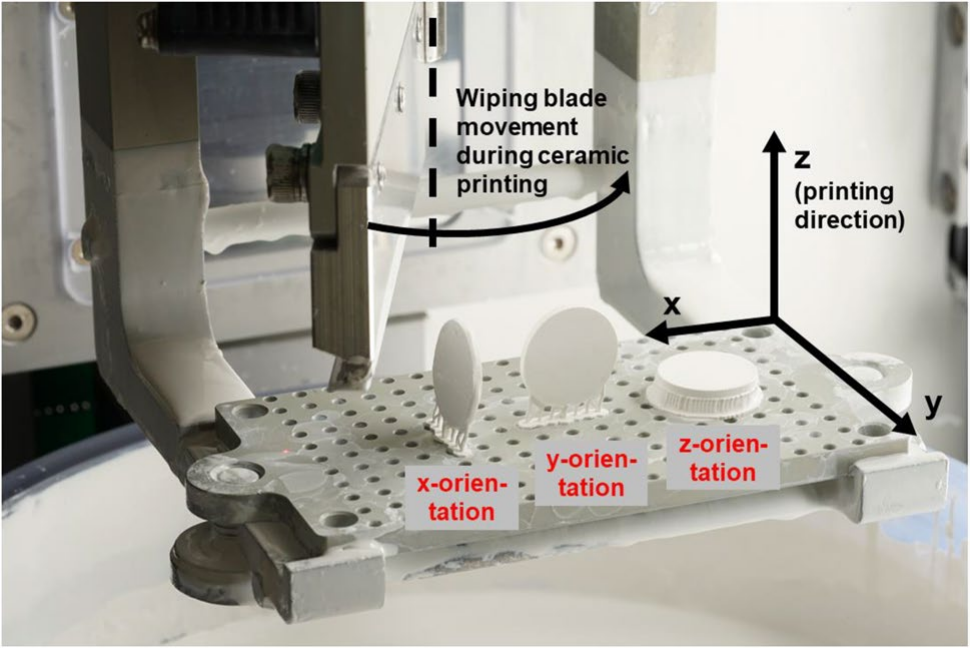
Coordinate system used with the AON ZIPRO-D printer. The face normal of the printed cylindrical discs was oriented in the x-, y- or z-direction. The z-direction is the printing direction and excess material for each new layer was swiped in the x-direction

A cylindrical specimen with a radius (*r*) of 6.2 mm and a width (*b*) of 1.6 mm was designed using CAD design software (Geomagic DesignX; 3D Systems) and an STL file was composed. After transferring the specimen geometry to a slicing software (ZiproS slicing software; AON), specimens were nested and scaled according to the manufacturer’s recommendations, then support structures added before 3D printing with the new ZD printer (AON Inc., Seoul, South Korea). Sampling is depicted in Fig. 1. In total, 80 specimens were printed by the ZD printer from zirconia slurry (InniCera BCM W1000; AON). Of these specimens, 60 were printed in a vertical orientation (printing layers perpendicular to the circular specimen surface, *n* = 40 with x-orientation, *n* = 20 with y-orientation) and 20 were printed in a horizontal orientation (z-orientation, printing layers in parallel with the circular specimen surface) (see Fig. 2 in addition which shows the three nesting orientations). ZD samples were debinded and presintered up to 1100 °C (debinding time: 30:05 h; ZIRFUR, AON) before being sintered to full density at 1500 °C for 5 h (HTCT 08/16; Nabertherm, Lilienthal, Germany). Half of the specimens with x-orientation (*n* = 20) were fabricated with staining and half (*n* = 20) without staining. Specimens were stained after debinding using the immersion method. Specimens were immersed for 1 min in the staining liquid (e.max ZirCAD Coloring liquid A3; Ivoclar Vivadent). Excessive color was drained and specimens were dried in a preheating furnace beginning at room temperature and ending at a temperature of 100 °C (Kavo EVL Type 5615; Kavo GmbH; Biberach, Germany). A further 20 specimens out of the entire sample *N* = 100 were printed with the LC printer in vertical nesting orientation (x-orientation) with a debinding/sintering time of 50 h (LithaCon 3Y 210 and CeraFab 7500; Lithoz; Vienna, Austria, furnace: HTCT 08/16; Nabertherm) (see Figs. 1 and 2). Zirconia samples fabricated with both printers consisted of 3-mol%-yttria-stabilized zirconia polycrystal material (3Y-TZP). The ZD and LC printers use digital light processing at wavelengths of 520 and 460 nm, respectively, to build up specimen layer-by-layer. When using ZD, the slurry vat is filled with at least 2 L of material dispersion. The objects are built on a perforated printing platform from the bottom to the top, whereby with each printing layer the platform is lowered by 50 μm into the vat and a squeegee flattens the slurry surface before selective light curing of each layer. This approach intended to promote torsion-free production of the object. The disadvantage here is that a lot of slurry has to be provided and the residual slurry has to be prepared again for the next printing job. In contrast, with LC, the objects are created upside-down. The platform with the already partly printed object hanging on its lower side is lowered into a vat with only a thin layer of slurry, generated by rotating the vat with applied slurry beneath a squeegee until a small gap of 25 μm remains. After selective light curing, the platform is raised and a new slurry layer can be created. Excess slurry was manually removed with an airbrush. According to the manufacturers’ instructions, isopropanol (purity ≥ 99.5%) was used to clean ZD samples and LithaSol 30 (Lithoz) was used to clean LC samples.

After sintering, the 1.6-mm-thick samples were ground and polished (MD Piano diamond disks, #220, #500, #1200; Struers, Willich, Germany) according to ISO 6872 requirements in a semi-automatic grinding and polishing device (Tegramin25; Struers). The specimen dimensions were then measured using a digital micrometer screw (MicroMar 40 EWR; Mahr, Göttingen, Germany). All samples met ISO 6872 requirements (1.0 mm < *b* < 1.4 mm) and noted for the individual biaxial strength calculation. Macroscopic flaws and voids were controlled in all specimens using digital microscopy (Smartzoom5, Zeiss, Jena, Germany).

### Biaxial flexural strength testing

**Fig. 3:**
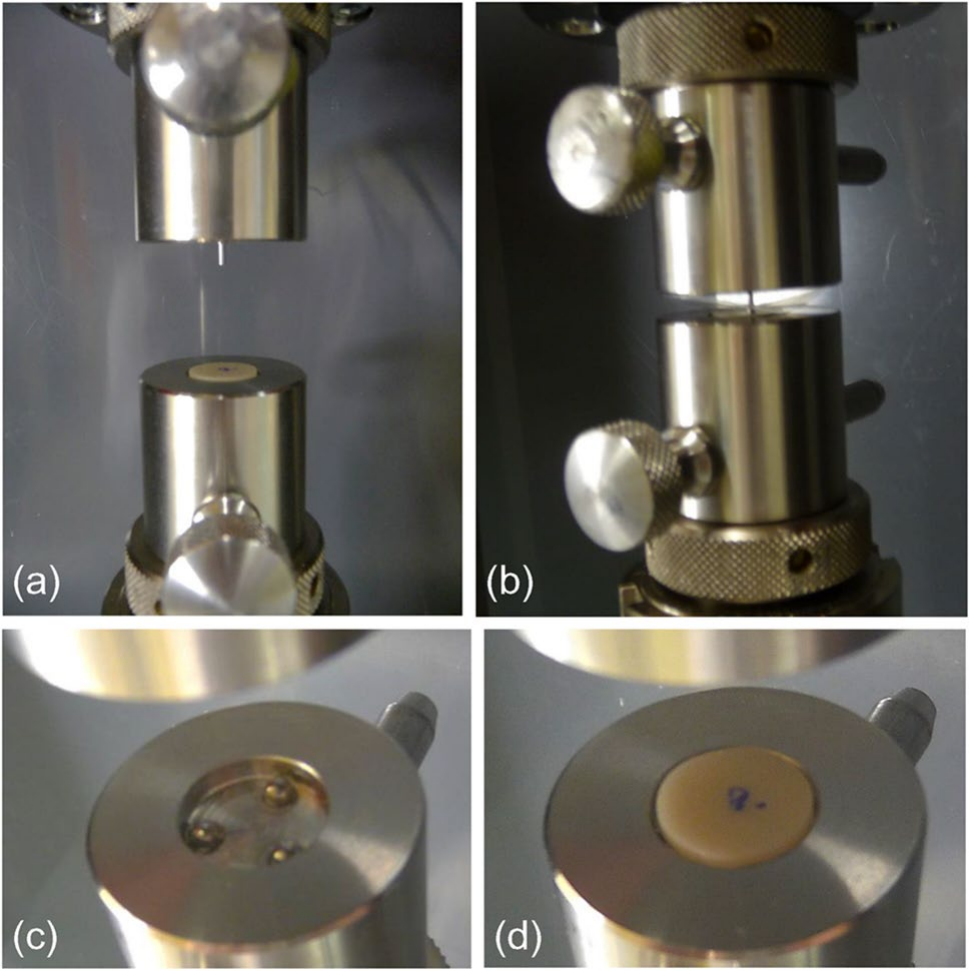
Test setup for the biaxial flexural strength test in the universal testing machine, **a** shows an overview, **b** detail picture of the test with polyethylene foils placed between sample and indenter and balls and sample, **c** shows the 3-ball support of the lower part without specimen, **d** with placed specimens. The cylindrical indenter is located in the upper part and is lowered to the specimen while testing

All specimens were tested to fracture (maximum load P) using a universal testing machine (Z005; ZwickRoell, Ulm, Germany) at a cross-head speed of 1 mm/min (see Fig. 3a–d). With a given setup and sample geometry, the individual biaxial strength values were computed as follows:

$$\begin{array}{l}\upsigma = -0.2387\mathrm{ P}\frac{{\text{X}}-{\text{Y}}}{{{\text{b}}}^{2}}\\ {\text{X}}=\left(1+\upnu \right){\text{ln}}{\left({{\text{r}}}_{2}/{{\text{r}}}_{3}\right)}^{2}+\left[\left(1-\upnu \right)/2\right]{\left({{\text{r}}}_{2}/{{\text{r}}}_{3}\right)}^{2}\\ {\text{Y}}=\left(1+\upnu \right)[1+{\text{ln}}{\left({{\text{r}}}_{1}/{{\text{r}}}_{3}\right)}^{2}]+\left(1-\upnu \right){\left({{\text{r}}}_{1}/{{\text{r}}}_{3}\right)}^{2}\\ \text{with} \\ \upnu=0.25 \;\; \text{assumed according to ISO 6872,}\\ {{\text{r}}}_{1}=5\;\text{mm,}\;{{\text{r}}}_{2}=0.6\;\text{mm,}\;{{\text{r}}}_{3}\text{: sample radius,}\;\text{b: sample thickness.}\end{array}$$

### Statistical analysis

All statistics were performed using SPSS version 28 (IBM; New York, USA). Mean values and standard deviations (SD) of the recorded flexural strengths were calculated for each group. Weibull distributions were fitted to the measured data and Weibull parameters (characteristic strength: σ_0_, Weibull modulus: m) were calculated. Variances differed between groups, so Welch ANOVA and Dunnet T3 post hoc tests were carried out to estimate the possible effects of material, nesting orientation, and staining on biaxial flexural strength. The assumed level for statistical significance was *α* < 0.05.

## Results

**Fig. 4:**
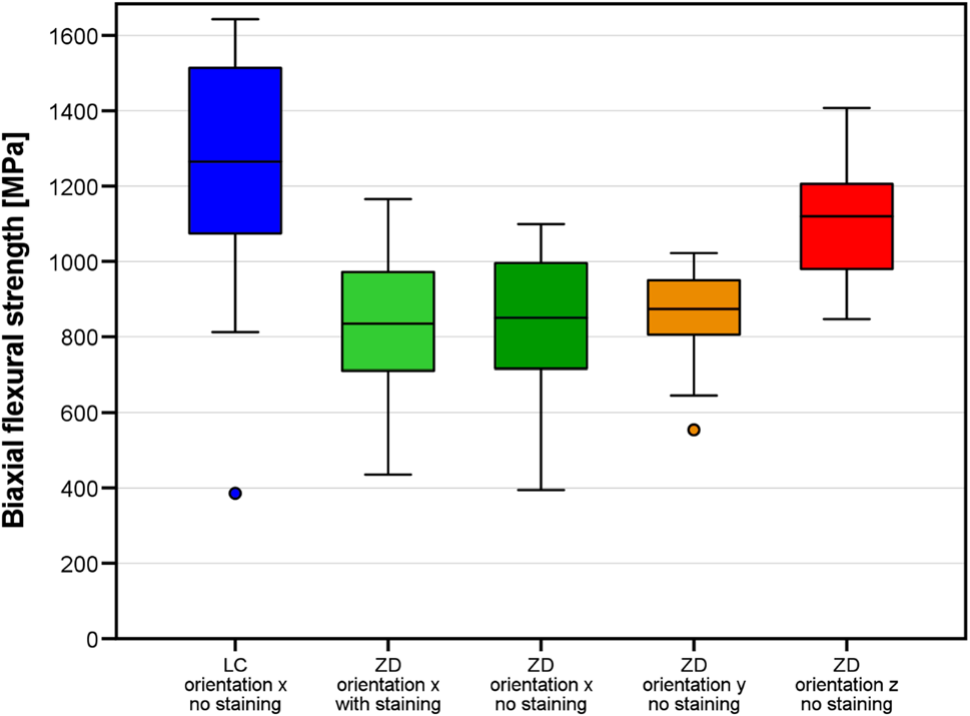
Boxplot diagram showing the biaxial flexural strength of the samples (LC: LithaCon 3Y 210, ZD: ZIPRO Dental) according to nesting orientation and staining

**Fig. 5:**
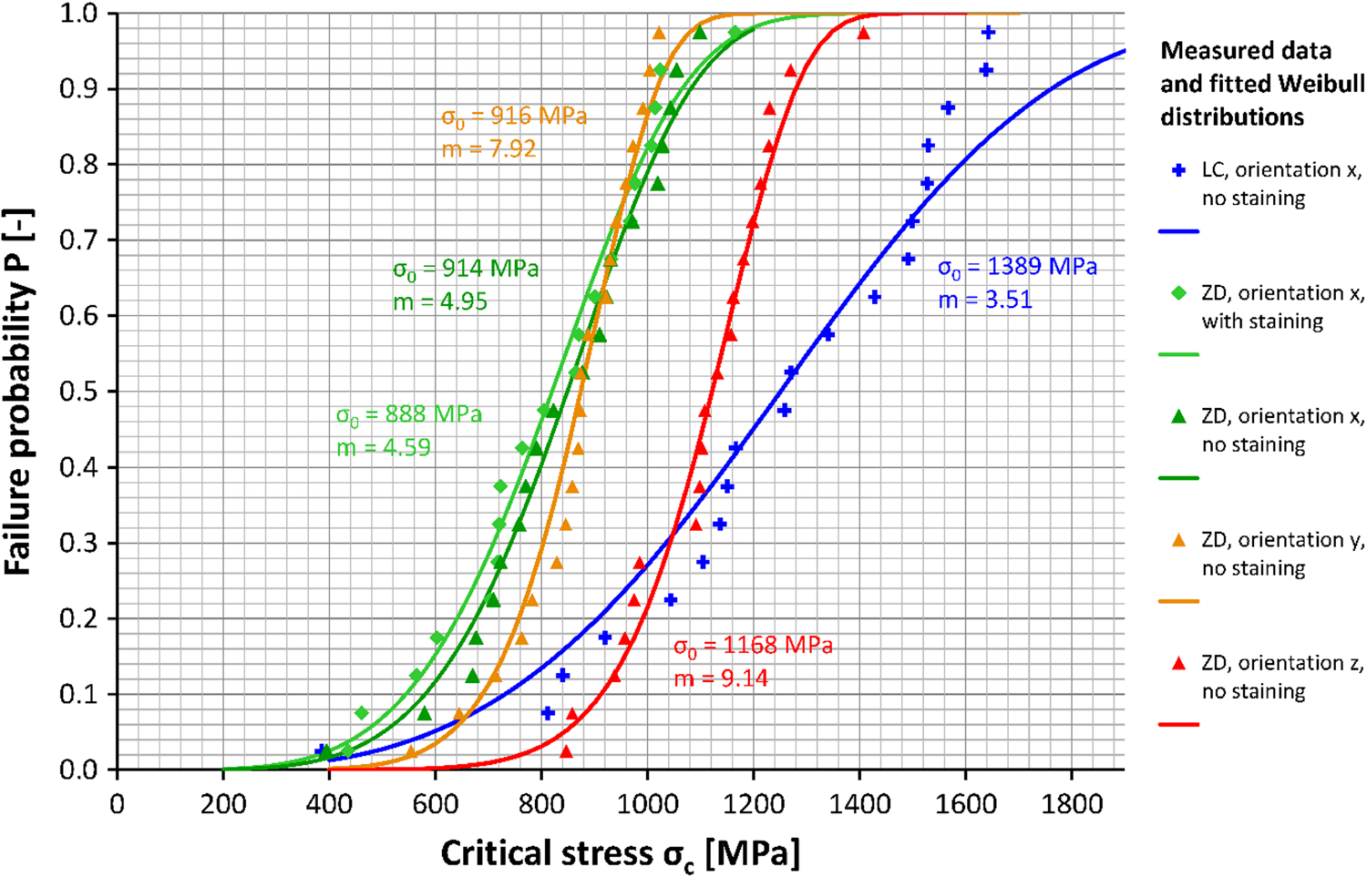
Correlation of failure probability P and critical stress σ_c_. Measured data as well as fitted Weibull distributions are displayed for the different groups

**Fig. 6:**
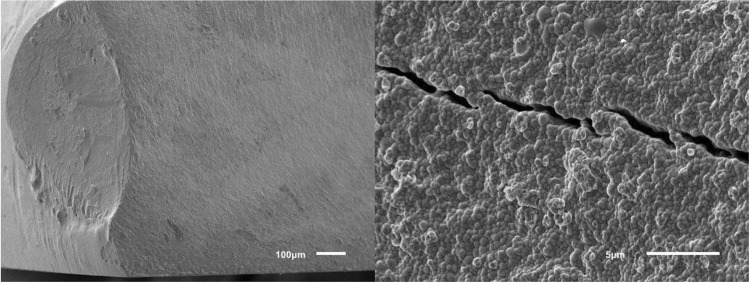
SEM images of representative LC samples at magnifications of 100 × (left) and 5000 × (right)

**Fig. 7:**
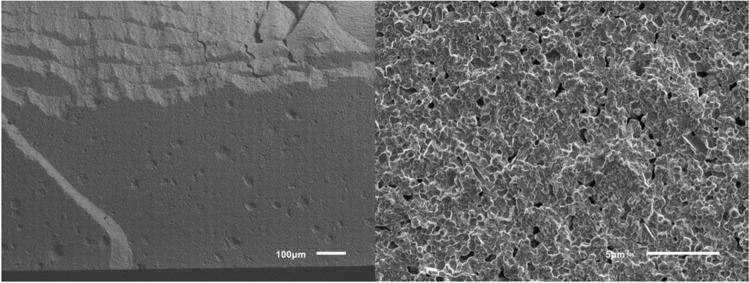
SEM images of representative ZD samples without staining and in the x-orientation of the face normal vector during 3D printing at magnifications 100 × (left) and 5000 × (right). ZD samples with y-orientation were similar to the images shown here

**Fig. 8:**
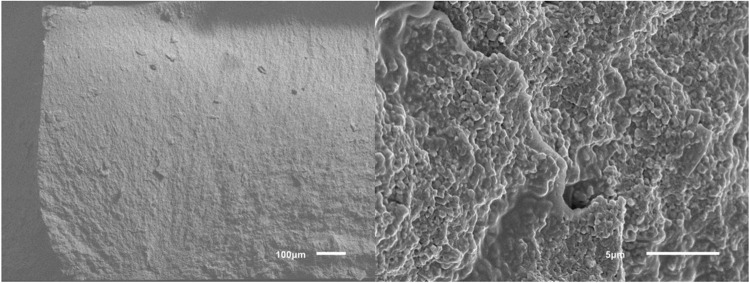
SEM images of representative ZD samples without staining and z-orientation of the face normal vector during 3D printing at magnifications 100 × (left) and 5000 × (right)

The results for the different groups are shown in Table 1 and Fig. 4. Significant differences in biaxial flexural strength between groups were found by Welch ANOVA. The biaxial flexural strength was highest in horizontally nested (z-orientation) ZD and LC samples (no difference between these groups, *p* = 0.659) with mean values of 1107 MPa and 1238 MPa, respectively. These specimens typically fractured into 4–5 shards. For ZD samples with x- and y-nesting orientations, the printing layer interfaces were the weakest link of the chain leading to significantly lower mean fracture strengths (811–862 MPa) than ZD samples with z-orientation and LC samples (*p* ≤ 0.001 for all pairwise tests). These samples typically fractured into two (sometimes three) pieces along their printing layer interfaces. Staining slightly lowered the strength (811 MPa vs. 838 MPa) of ZD samples printed with x-orientation but this effect was not significant (*p* = 1.000). There were also small and insignificant differences between the x and y nesting orientations (*p* = 0.976), but it should be noted that samples with y-orientation had a lower data variability (lower SD, higher Weibull modulus). Figure 5 shows that the Weibull distributions fitted well with the measured data. For a low failure probability of *p* = 0.05 = 5%, ZD samples with x-orientation were correlated with a critical stress value of σ_c_ ≈ 450–500 MPa, LC samples and ZD samples with y-orientation with a critical stress value of σ_c_ ≈ 600–620 MPa and ZD samples with z-orientation with the highest critical stress value of σ_c_ ≈ 630 MPa. SEM showed LC and ZD samples with z-orientation did not fracture along their printing layer interfaces in contrast to ZD samples with x- or y-orientation (Figs. 6, 7 and 8). We also showed that ZD samples had a higher porosity than LD samples, especially along the printing layer interface. Zirconia grain size was similar for both materials.

**Table 1:** Biaxial flexural strength of specimens in the different groups. Respective parameters are provided for normal distribution (mean value, standard deviation) and Weibull distribution (characteristic strength σ_0_, Weibull modulus m). Different uppercase letters indicate significant differences between the groups

Material	Orientation	Staining	*n* [-]	Shards/sample n_s_ [-]		Flexural strength [MPa]		Weibull parameters	
				Mean value	SD	Mean value	SD	σ_0_ [MPa]	m [-]
ZD	x, ZD1	Yes	20	2.1	0.3	811^A^	197	888	4.59
	x, ZD2	No	20	2.2	0.5	838^A^	182	914	4.95
	y, ZD3	No	20	2.2	0.4	862^A^	122	916	7.92
	z, ZD4	No	20	4.2	0.6	1107^B^	144	1168	9.14
LC	x	No	20	4.7	1.5	1238^B^	327	1389	3.51

## Discussion

Our results suggest that staining of printed zirconia did not affect flexural strength (rejection of hypothesis 1 while the factors nesting orientation (only varied with ZD) and material did affect flexural strength (acceptance of hypotheses 2 and 3).

The flexural strength of LC samples observed in this study were consistent with those reported in a previous study [16]. However, flaws, voids or delaminations can occur between the layers of printed zirconia [10, 11, 16], and these problems may be enhanced by post-processing of the material [11, 17, 20, 21]. To standardize the workflow, all materials in this study were used in accordance with the manufacturer’s instructions. For LC samples, prefabricated cartridges were used directly in the 3D printer, whereas for ZD samples, the slurry had to prepared and mixed before use. Furthermore, the LC printer prepares a thin film of zirconia during each step, onto which the object is lowered until a 25-μm gap (printing layer thickness) is formed with the platform. In contrast, the ZD printer lowers the object into the slurry vat so that slurry flows over the object; after this, the slurry is wiped off with a squeegee to create a film thickness of 50 μm. Our results suggest that this technique might favor small air pockets in the ZD printing slurry, creating voids and increasing porosity in the sintered zirconia. When comparing the facture modes of vertically nested specimens, almost all ZD specimens fractured along the layer interfaces, whereas no favored fracture directions could be observed for LC specimens. This might be caused due to more air inclusions at the interfaces of ZD specimens (Fig. 7). Since fracture surfaces of horizontally nested specimens did not show such porosity, we assumed that this problem was restricted to the layers’ seams. With LC, each cured layer sticks to the bottom of the vat and has to be pulled off before the next layer can be generated. This is not necessary with the bottom-to-top building direction of the ZD printer. Thus, in use of ZD, only the free slurry surface is light-cured, theoretically leading to less object torsions. In this study, the default light curing parameters were used. A possible impact of varied lighting duration/intensity in terms of improving layers’ interfaces should be the topic of future studies. For the sake of completeness, it should be kept in mind that slurry provision is more extensive in use of ZD. In order to minimize slurry consumption, slurry remaining in the vat has to be prepared again prior to the next printing job making the approach time consuming and technique sensitive. Back to facture strengths, cleaning and debinding were more conservative for LC than for ZD, with LC using a low percentage isopropanol while ZD uses nearly pure isopropanol. Liebermann et al. showed that cleaning procedures can degrade the material [17]. Furthermore, the debinding and firing time for ZD was roughly 70% of that for LC. SEM images (Figs. 6, 7 and 8) revealed voids and/or flaws in both LC and ZD samples, in agreement with previous literature [11, 16, 21], but porosity and flaws were more prominent in the printing layer interfaces of ZD samples as described above. These flaws can initiate cracks, which might cause failure at rather low (nominal) tensile stress values [11]. In our study, ZD samples with tensile stresses acting perpendicular to printing layer interfaces (x- and y-orientation) had only 70% of the strength of samples with layers parallel to the tensile stress direction (z-orientation). This finding, together with the fact that x- and y-oriented samples typically fractured in two halves along the printing layer interfaces, indicates that the layer interfaces are the weak spot of ZD-printed zirconia. Internal pilot studies have shown that the strength of LC samples is not affected by nesting orientation, which is why LC samples were only tested with one nesting orientation (x-orientation). Both LC and ZD samples showed a mean biaxial flexural strength of > 500 MPa, making them at least class 4 materials according to ISO 6872 standards. For clinical success, small failure rates are needed. Our results indicate similar critical stress values between 500 and 600 MPa correlating with a failure rate of 5% for LC and ZD samples. Since mean strength in all test groups was > 800 MPa, LC and ZD also fulfill the prerequisites of class 5 materials. Due to the high variability, such a recommendation should be handled with care. Importantly, the cylindrical discs used in this study have a very favorable shape for debinding. In dental restorations with sharp edges (such as fissures on the occlusal surfaces) and thick objects (such as pontics), debinding might create more flaws compared with the discs. Further studies are needed to show the performance of real 3D-printed restorations.

With real crowns, high tensile stresses occur at the occlusal surface next to contact points and at the inner crown surface beneath contact points if the wall is not thick. The tensile stress is highest parallel to the crown surface; therefore, a horizontal orientation of the occlusal surface is favorable. However, high tensile stress will never be limited to one location because of complex mechanical factors, such as the geometry and loading of dental restorations. Therefore, material anisotropy cannot be completely solved by simply choosing an appropriate nesting orientation. Further investigations should find ways to improve ZD printing as well as debinding and sintering protocols.

Zirconia staining is frequently used to determine the color of zirconia when fabricating monolithic restorations. Staining of milled zirconia has not been shown to affect restoration strength in previous studies [22]; this has been shown previously for LC restorations [16] and now for ZD restorations in the present study.

## Conclusions

All 3D-printed zirconia samples exceeded the required mean flexural strengths for class 5 restorations according to ISO 6872 standards. While the flexural strength of ZD-printed zirconia in the vertical orientation was lower than that of LC samples, the ZD printer showed at least comparable reliability. Further optimizations such as modification of light intensity/duration and preparation of the slurry should be investigated in further studies.

## Data Availability

Source data will made available on request to corresponding author.
